# Bowel wall thickening and hyperemia assessed by high-frequency ultrasound indicate histological inflammation in Crohn’s ileitis

**DOI:** 10.1007/s00261-020-02895-8

**Published:** 2020-12-24

**Authors:** Ruediger S. Goertz, Stefanie Hensel, Dane Wildner, Markus F. Neurath, Deike Strobel

**Affiliations:** 1 Department of Internal Medicine 1, Friedrich-Alexander-Universität Erlangen-Nürnberg (FAU), Ulmenweg 18, 91054 Erlangen, Germany; 2 Department of Internal Medicine, Krankenhäuser Nürnberger Land GmbH, Simonshofer Str. 55, 91207 Lauf, Germany

**Keywords:** Crohn’s disease, Ultrasound, Bowel wall, Inflammation, Histology

## Abstract

**Purpose:**

We evaluated ileal bowel wall thickness and semiquantitative vascularization by ultrasound in correlation with the presence or absence of histopathological inflammation in patients with Crohn’s disease (CD).

**Methods:**

We conducted a retrospective analysis of 221 ultrasound examinations of the terminal ileum or neoterminal ileum in CD patients with biopsies of the ileum during colonoscopies within 8 weeks of the ultrasound. Ultrasound data were obtained from an inflammatory bowel disease ultrasound register from 2011 to 2017. Bowel wall ultrasound was performed by a high-frequency, linear transducer (7–12 MHz). Presence of bowel wall thickening (> 3 mm), vascularization by the Limberg score, and presence of ileal histopathological inflammation were analyzed.

**Results:**

In 221 bowel wall ultrasound examinations of CD patients (128 female, 93 male, mean age 37.5 years), a thickened bowel wall was found in 140 (63.3%) and hypervascularization (corresponding to a Limberg score ≥ 2) in 96 (43.4%) cases. In 187 (84.6%) cases, ileal inflammation was confirmed by histopathology and in 34 (15.4%) cases no inflammation was shown. Bowel wall thickening showed a sensitivity of 70.1%, a negative predictive value (NPV) of 30.9%, a specificity of 73.5% and a positive predictive value (PPV) of 93.6% for the detection of histopathological ileal inflammation. Hypervasularization had a low sensitivity (49.7%) and NPV (24.8%), but high specificity (91.2%) and PPV (96.9%).

**Conclusion:**

In this CD subcohort of an ultrasound register, pathologic ultrasound findings were quite common. Bowel wall thickening (> 3 mm) and hypervascularization are good predictors of histopathological inflammation within the terminal ileum or neoterminal ileum. Normal ultrasound findings without bowel wall thickening and without hypervascularization do not rule out histopathological inflammation.

## Introduction

Diagnosis of Crohn’s disease (CD) is based on the clinical appearance and course of the disease as well as a combination of endoscopic, histological, sonographic/radiological and laboratory data [1]. During follow-up of patients with CD or search for extramural complications, ultrasound of the abdomen and the bowel wall is regularly an easy diagnostic step [2, 3]. High-frequency Ultrasound identifies wall thickening of the small bowel or the colon as well as the extent and location of inflammation. Furthermore, it can detect complications such as lymph nodes, ascites, mesenteric fat tissue or abscesses, fistulas and stenosis [4]. For bowel wall thickening a cut-off of > 3 mm is recommended for the detection of disease activity in CD with high sensitivity (up to 89%), while a cut-off of > 4 mm serves for better specificity (up to 98%) [1, 5]. Color Doppler ultrasound evaluates hypervascularization of the affected bowel wall segment semiquantitatively and correlates with disease activity [6, 7].

Ultrasound is cheap, easily available, comfortable for the patients, and can evaluate and monitor transmural healing [8]. Colonoscopy with biopsy, in contrast, is accompanied by purge, sedation, and possible complications such as bleeding or perforation. During endoscopy forceps biopsy for histopathological workup is important for the diagnosis of CD and serves as a standard for the assessment of disease activity and treatment response [9, 10]. There is no consensus about the exact histological parameters to stage disease activity [11]. European guidelines also mention novel ultrasound imaging techniques such as contrast-enhanced ultrasound (CEUS) and sonoelastography for possible use in CD [1, 8]]. CEUS overcomes the limitations of color Doppler (impossible in cases of slow blood flow in small vessels in deep-lying bowel wall segments) and improves the detection of hypervascularity and perfusion. CEUS has high accuracy in the detection of active disease, for diagnosis of postoperative CD recurrence or treatment outcome and help to differentiate between inflammatory and fibrotic strictures. Sonoelastography may be applied to evaluate the stiffness of a Crohn´s stenosis, although underlying data are limited and acquisition methods are still unstandardized.

Data on ultrasound of the bowel wall in CD patients to predict histological inflammation are sparse. In a study of 32 CD patients evaluating ileal Doppler sonography and results of the ileocolonic biopsy, promising results were presented: a Limberg score ≥ 1 using a 4 mm cut-off of bowel wall thickening predicted histological disease activity with high sensitivity and a high positive predictive value [7]]. Our retrospective study aimed to evaluate ultrasound findings such as bowel wall thickening and hypervascularization in patients with CD to detect histological inflammation of the terminal ileum or neoterminal ileum.

## Materials and methods

The retrospective study was performed at our Gastroenterology Department of Internal Medicine. The data were acquired from a register of ultrasound investigations of patients with inflammatory bowel disease (CD or ulcerative colitis). Patients with indeterminate colitis or non-classified colitis were not considered. The register includes outpatient visits of patients and hospitalized patients. The number and frequency of ultrasound examinations of a patient depended on the course of CD with disease activity and therapy. All patients included in our study were aged ≥ 18 years with any medication.

**Fig. 1:**
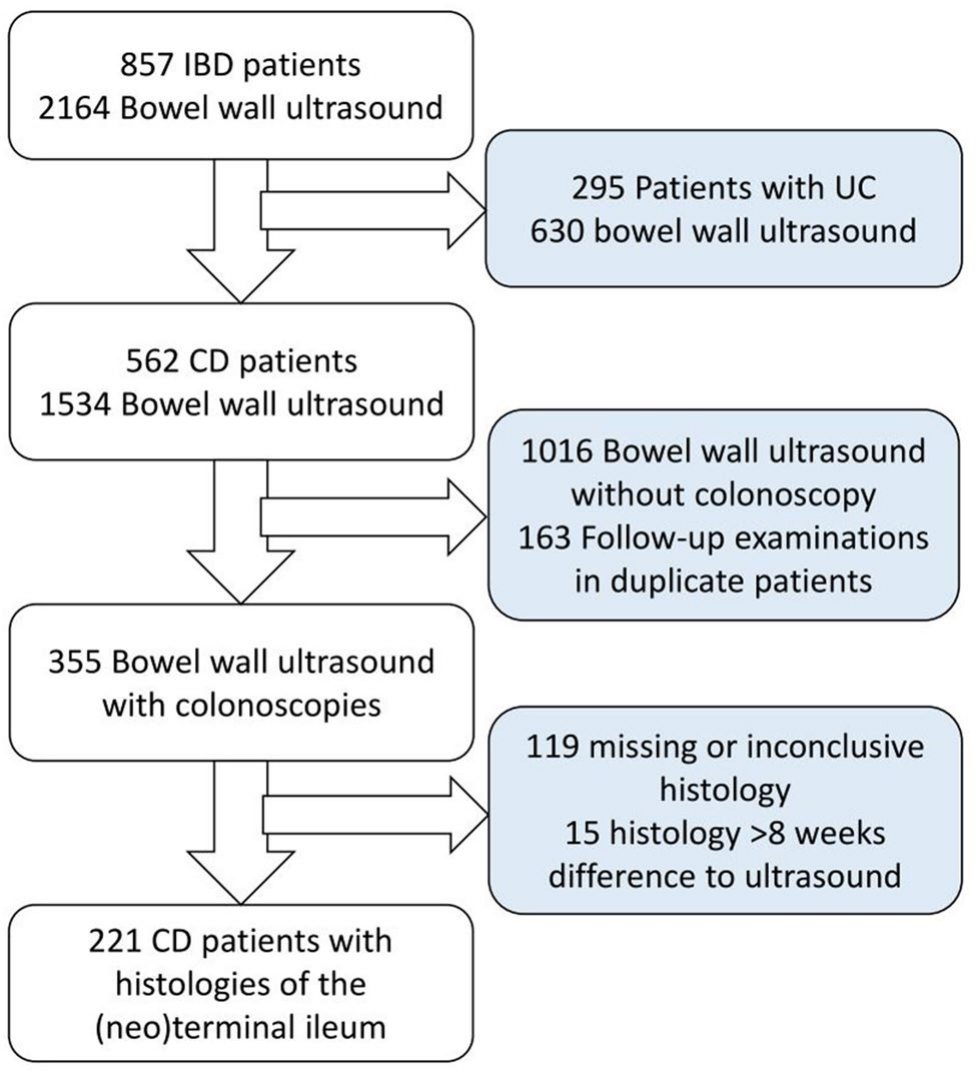
Flow chart of a retrospective analysis of 221 ultrasound examinations in CD from a register of ultrasound in inflammatory bowel disease. CD Crohn’s disease, UC ulcerative colitis

In our study, we analyzed ultrasound examinations of the bowel in patients with proven CD between 2011 and 2017. Exclusion criteria were diagnosis of ulcerative colitis, missing colonoscopy with ileal biopsies, duplicate patients and a difference of more than 8 weeks between the date of colonoscopy and the ultrasound examination (Fig. 1). Possible indications for ileal biopsies were proof of CD diagnosis, staging before initial treatment, unclear deterioration of inflammation, monitoring therapy outcome or detection of mucosal healing. The local ethics committee approved the retrospective analysis (Re-No. 41_13B). Using PACS analysis, the results of the ultrasound examinations, endoscopic and histologic findings as well as patient data and laboratory data (C-reactive protein (CRP)), were collected only from the CD patients. The Harvey–Bradshaw–Index (HBI)—calculated for assessing disease activity clinically using scores of general well-being, the severity of abdominal pain, the number of non-solid stools per day, and the presence of an abdominal resistance or complications—was transferred from the report [12].

**Fig. 2:**
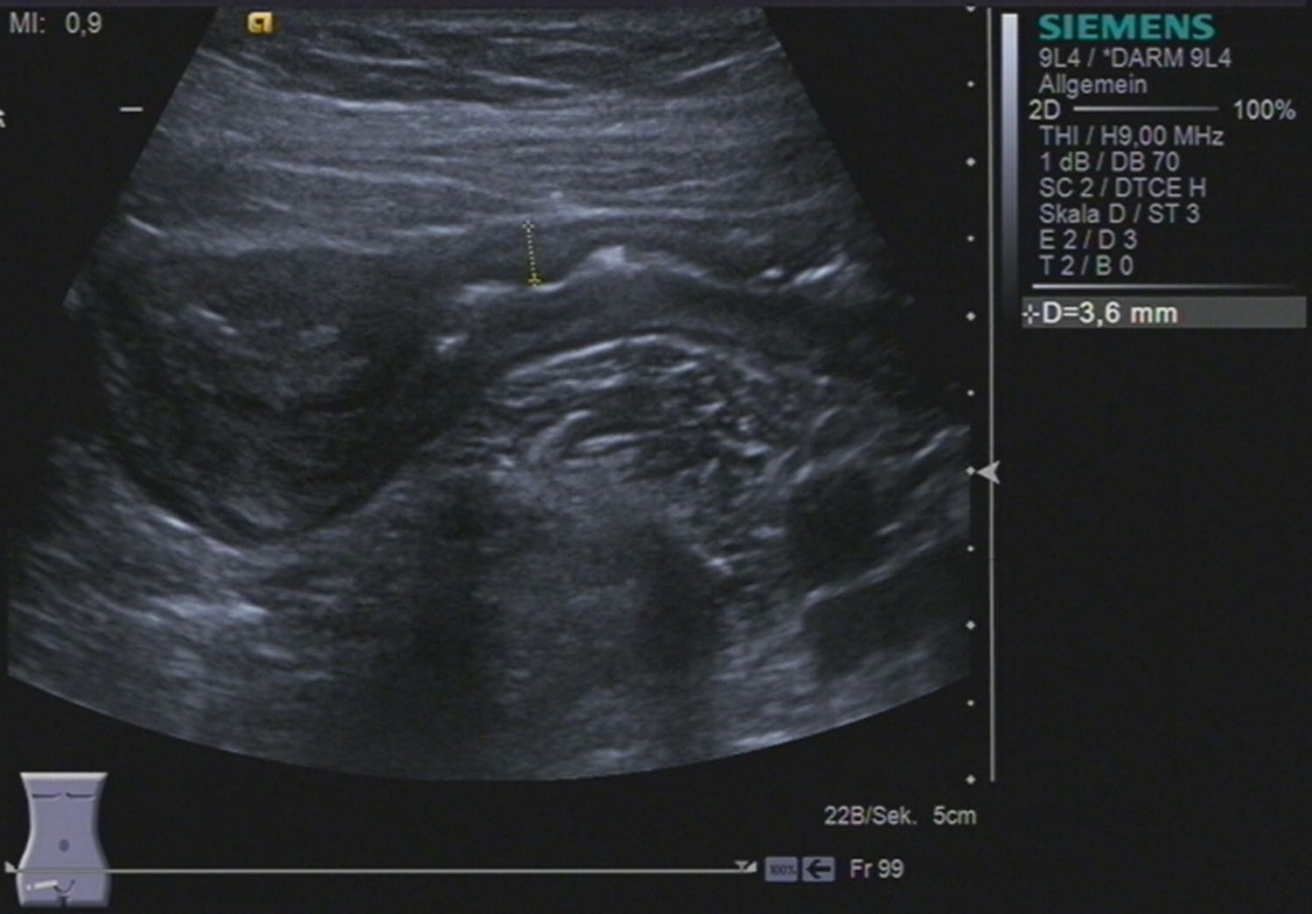
Sonographically active Crohn’s ileitis with 3.6 mm bowel wall thickening and a Limberg score of 3

**Fig. 3:**
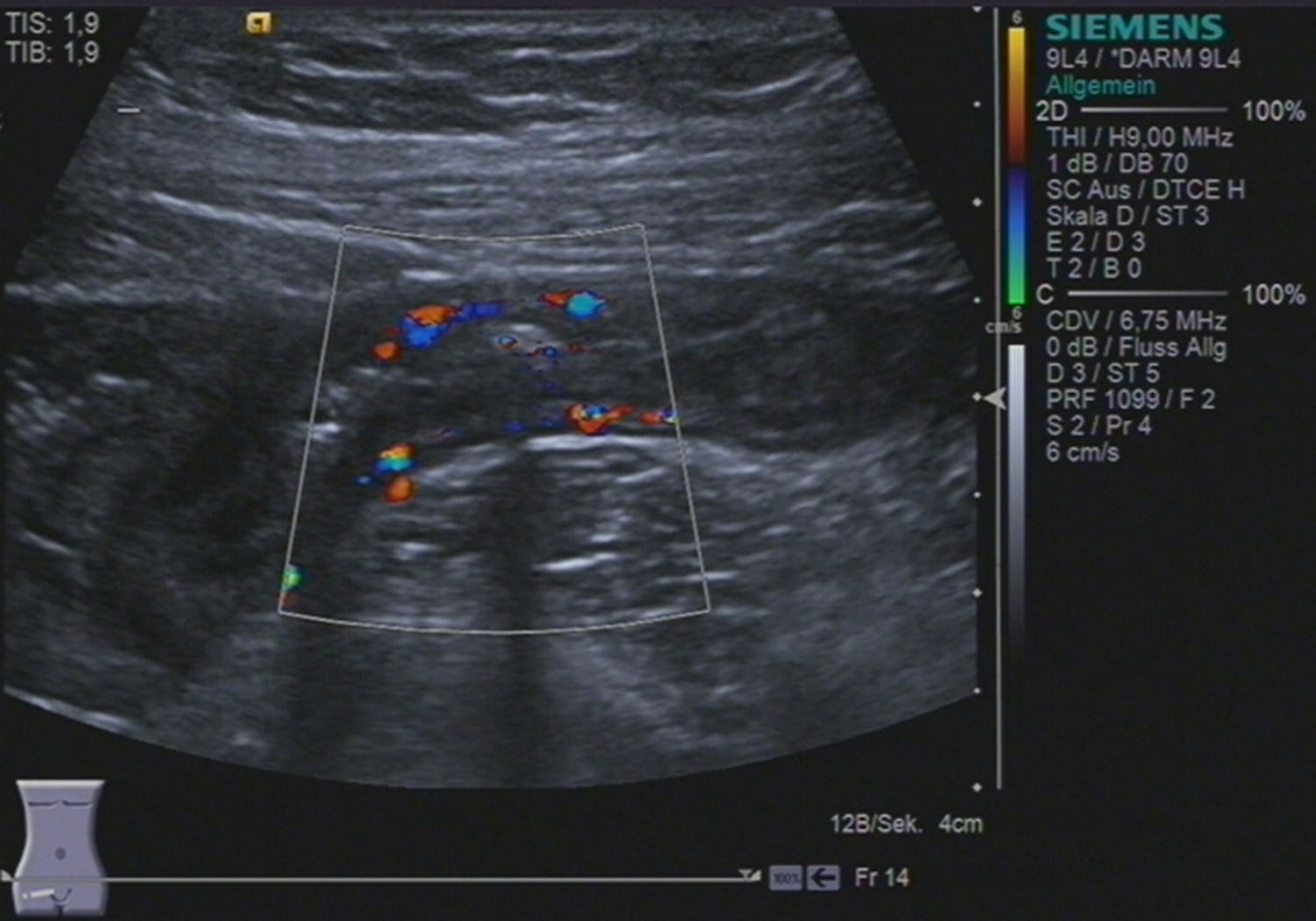
Sonographically active Crohn’s ileitis with 3.6 mm bowel wall thickening and a Limberg score of 3

**Fig. 4:**
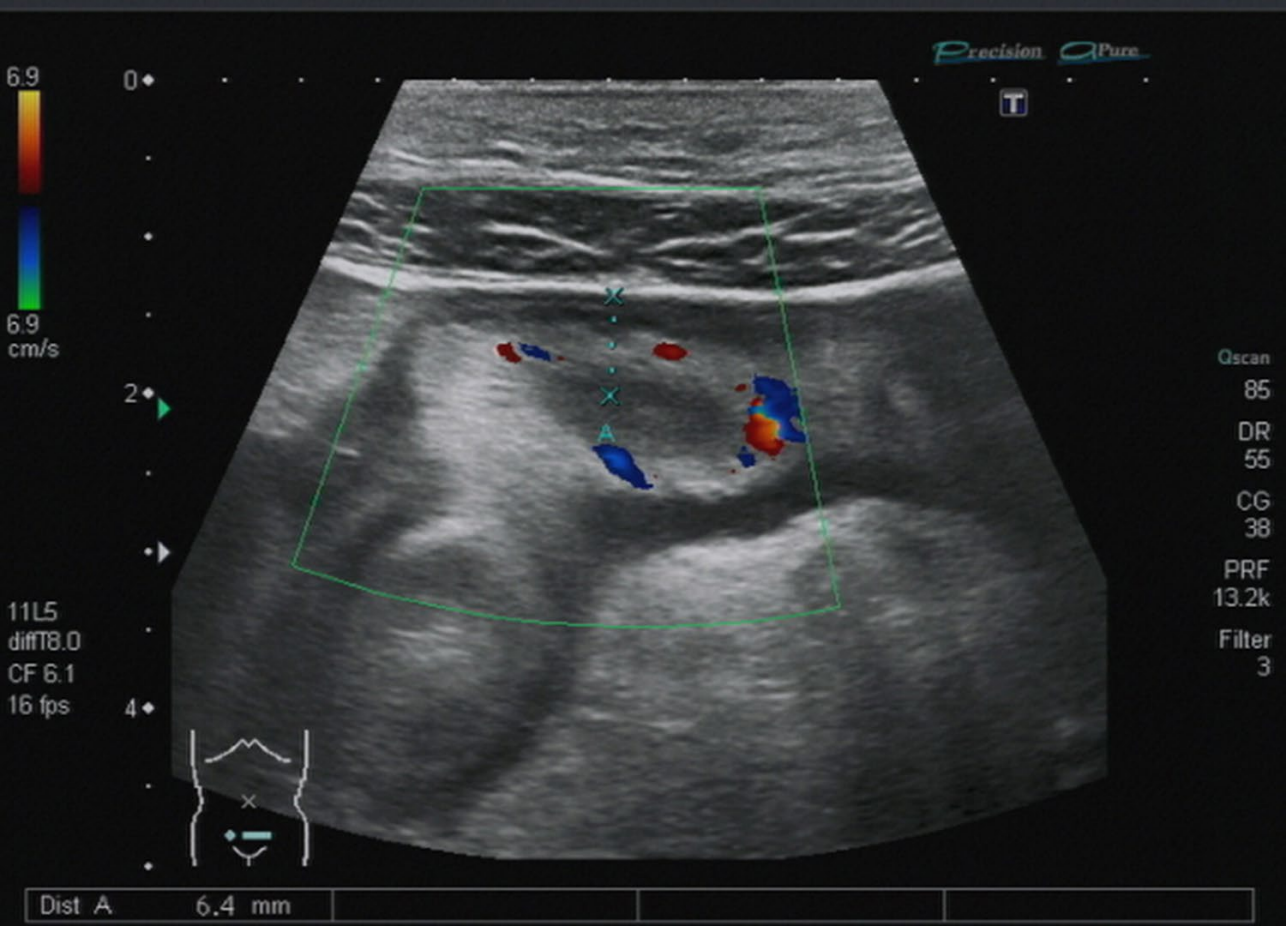
Ileal bowel wall thickening (6.4 mm) with a Limberg score of 2

For bowel wall ultrasound with a high-frequency transducer, different high-end ultrasound systems such as Siemens S2000, Toshiba Aplio 500 and GE Logiq E9 were used in this study. Different senior physicians with more than eight years ‘ultrasound experience performed high-frequency ultrasound. The ultrasound examination of CD patients was standardized. Starting with a convex probe (2–6 MHz) the large and the small bowel were visualized to improve orientation. Landmarks for evaluation of the terminal ileum were the right iliac vessels and psoas muscle. Wall thickness and vascularity were assessed using the linear transducer (7–12 MHz) (see Figs. 2, 3 and 4) [13]. The dynamic range for B-mode ultrasound was 65–85. Color Doppler pulse repetition frequency was 950–3000 Hz (~ 6–19 cm/s) with a frequency between 3.6 and 6.75 MHz depending on the sensitivity of the ultrasound machine used. Bowel wall thickening was measured by including the three layers (hypoechoic-hyperechoic-hypoechoic) corresponding to the mucosa, submucosa and muscular layer of the bowel wall and was defined with a > 3 mm threshold, and for further analysis, a > 4 mm threshold [1]. Ultrasound result often stated “normal bowel wall thickness” without exact figures, therefore thickness was deemed to be ≤ 3 mm. The degree of vascularization was classified semiquantitatively according to an adapted Limberg score [6]: 0 = no wall thickening, no color Doppler signal, ≥ 1 meant wall thickening with 1 = no color Doppler signal, 2 = dots of color Doppler signals, 3 = longer stretches of color Doppler signals, 4 = strong color Doppler signals with presentation within the mesenteric tissue (see Figs. 3 and 4). Singular cases of color Doppler signal in a normal thickened wall were grouped according to color Doppler grades 2–4. Surrounding pathologic findings such as ascites, lymph nodes, abscess, fistula, or mesenteric inflammation were also evaluated.

Colonoscopies within 8 weeks before or after an ultrasound of the bowel were included. If one or more biopsies were taken from the ileal bowel segment, these were sent to the Institute of Pathology for histopathological work-up. Histologically, the ileal mucosa was evaluated as non-inflammatory or with the presence of signs of inflammation depending on the presence of leucocyte infiltration, granulomas, erosion, ulcerations or disturbance of crypt architecture [14–16]. Each ultrasound examination was compared to the corresponding original histopathological report separately.

For statistical analysis the Statistical Package for the Social Sciences (version 24.0.0.2, IBM Corp., Armonk, NY) was used. Clinical, laboratory and ultrasound parameters were expressed as mean ± standard deviation. The range was shown in square brackets. Sensitivity, specificity, positive predictive value (PPV) and negative predictive value (NPV) were calculated. The Pearson coefficient was used for correlation analysis and, the t-test to compare mean values. Results were considered statistically significant for *p < 0.05 and statistically highly significant for **p < 0.001. All reported p values are two-sided.

## Results

There were 857 patients with inflammatory bowel disease in the register who had 2164 bowel wall ultrasound examinations carried out between 2011 and 2017, of which 562 were CD patients with 1534 bowel wall ultrasounds (Fig. 1). 1016 ultrasound examinations were excluded due to missing related colonoscopy. From the remaining 518 ultrasounds 163 repeated examinations in the same patient (follow-up) were excluded, although the baseline evaluation remained included. Another 134 examinations were excluded because of missing or inconclusive ileal histology (*n* = 119) or a time difference higher than 8 weeks to the previous or subsequent ultrasound (*n* = 15). Finally, 221 patients with 221 bowel wall ultrasounds and coherent, conclusive histology of the (neo)terminal ileum were included in the final analysis. The median time difference between ultrasound and colonoscopy was 8 days.

The 221 ultrasound examinations with colonoscopic ileal histology corresponded to 128 female and 93 male CD patients with a mean age of 37.5 years and with a clinically scored (HBI) mild to moderate disease activity (Table 1). CD duration was about 11 years. 73% of the patients were assessed during outpatient visits and 47% had undergone a previous bowel surgery. Ultrasound showed mesenteric fat inflammation, lymph nodes, stenosis, or ascites in decreasing frequency (33% to 6.8%) (Table 2). In 221 ultrasound examinations, data were missing for disease duration (*n* = 5), HBI (*n* = 56) and CRP (*n* = 26). Bowel wall thickening and an increased Limberg score were shown in 63.3% and 65.6%, respectively. The Limberg score did correlate statistically significantly with histopathological signs of inflammation within the (neo)terminal ileum (*r* = 0.379, *p* < 0.001), but not with the HBI (*r* = 0.141, *p* = 0.071). The proportion of pathologic bowel wall thickening, the Limberg score, and the HBI are higher in examinations in hospitalized patients than during outpatient visits.

**Table 1:** Overall characteristics of 221 Crohn’s disease bowel wall ultrasounds with histology of the (neo)terminal ileum

Crohn’s disease	All	Ileal histology of subcohort	
		Inflammation	No inflammation
Number of patients	221	187	34
Sex [female/male]	128/93	106/81	22/12
Mean age [years]	37.5 ± 14 [18–82]	37.1 ± 13.8	38.5 ± 15
Disease duration [years]	11.1 ± 10.8 [0–45] (*n* = 216)	11.6 ± 11.2	8.3 ± 7.5 (n.s.)
Previous Surgery	104 (47%)	96 (51.3%)	8 (23.5%)
Outpatient/hospitalized	162 (73%)/ 59 (27%)	137 (73%)/ 50 (27%)	25 (74%)/ 9 (26%)
HBI	7.9 ± 5.3 [0–30] (*n* = 165)	7.8 ± 5.1	8.4 ± 6.6
CRP (mg/l)	24.6 ± 40 [0.08–285.9] (*n* = 195)	24.1 ± 39	27.5 ± 46
Macroscopic ileal inflammation by colonoscopy	59 (67%) (*n* = 88)	74 (84%)	14 (16%)

**p < 0.001

*HBI* Harvey–Bradshaw–Index, *CRP* C-reactive protein

**Table 2:** Ultrasonic findings of ileal bowel wall ultrasound in Crohn’s disease (*n* = 221)

Crohn’s disease	All	Ileal histology	
		Inflammation	No inflammation
Number	221	187 (84.6%)	34 (15.4%)
Bowel wall thickening > 3 mm	140 (63.3%)	131 (70.1%)	9 (26.5%)
Bowel wall thickening > 4 mm	110 (49.8%)	105 (56.1%)	5 (14.7%)
Limberg score	1.4 ± 1.3 [0–4]	1.5 ± 1.3	0.4 ± 0.7** [0–2]
0	76	51	25
1	49	43	6
2	54	51	3
3	27	27	0
4	15	15	0
Limberg score ≥ 1	145 (65.6%)	136 (72.7%)	9 (26.5%)
Mesenteric inflammation	73 (33%)	66 (35.3%)	7 (20.6%)
Lymph nodes	43 (19.5%)	41 (21.9%)	2 (5.9%)
Ascites	15 (6.8%)	13 (7.0%)	2 (5.9%)
Stenosis	23 (10.4%)	20 (10.7%)	3 (8.8%)
Fistula	8 (3.6%)	8 (4.3%)	0
Abscess	2 (0.9%)	2 (1.1%)	0

**p < 0.001

Macroscopic findings of the terminal ileum by colonoscopy were available in 88 patients and showed inflammation in 67%. Histology showed inflammation in 84% of patients (Table 1). In particular, 34 (39%) of these 88 patients had normal ultrasound bowel wall measurements within the ileum, but colonoscopy revealed macroscopically visible inflammation in 12 of these cases and in all 12 patients histologic inflammation was finally proven.

**Fig. 5:**
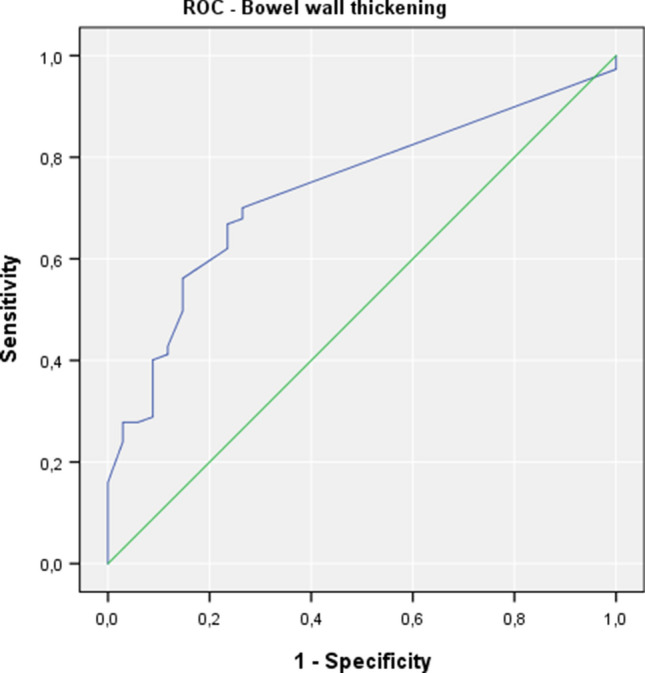
ROC analysis for the detection of histopathological ileal inflammation in Crohn’s disease by bowel wall thickening

**Fig. 6:**
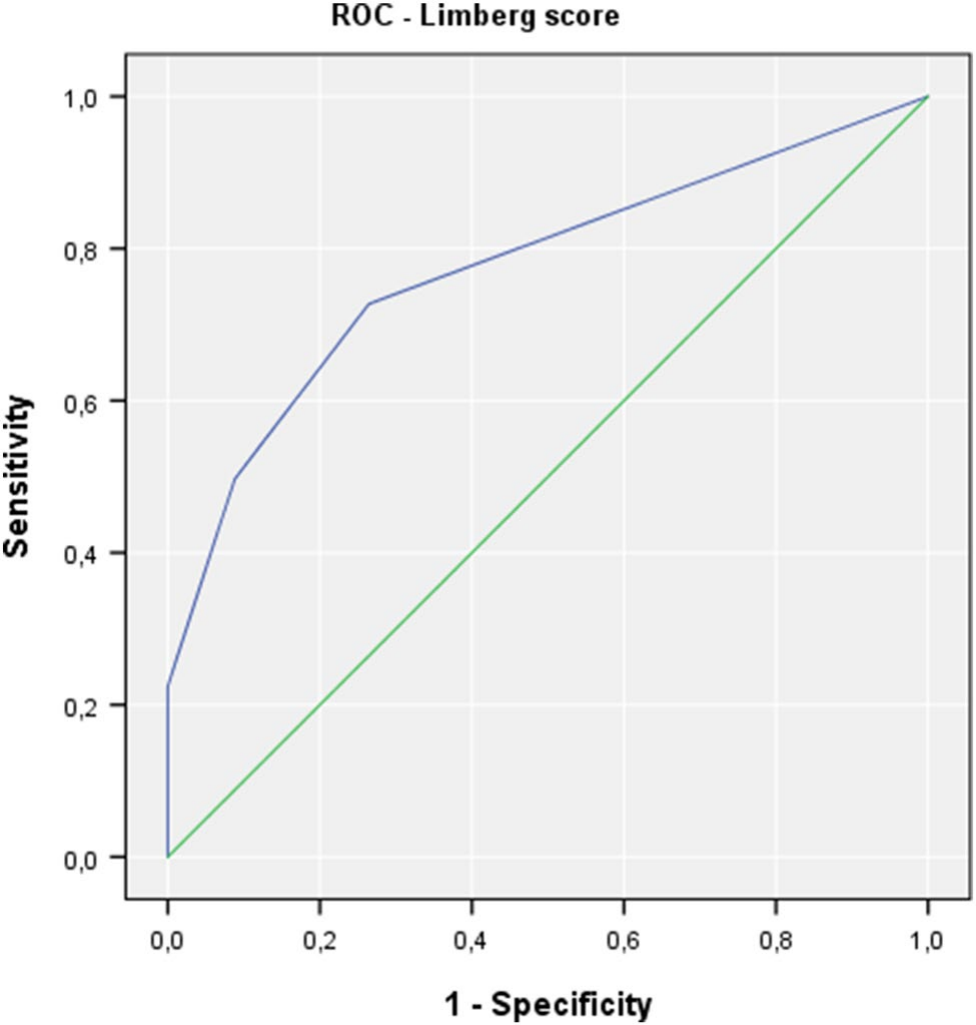
ROC analysis for the detection of histopathological ileal inflammation in Crohn’s disease by the Limberg score

In 187 from the 221 (84.6%) patients, ileal biopsies showed signs of histopathological inflammation within the (neo)terminal ileum, whereas 34 patients had no inflammation in the histopathological workup. Where wall thickening (> 3 mm) measured by ultrasound was present, 93.5% of patients had histological inflammation confirmed (Table 3). Patients with normal bowel wall measurement (≤ 3 mm) had histological inflammation in 69.1% of cases. HBI and CRP-level showed no statistical difference between those with histological inflammation and those without. The proportion of patients with previous surgery, bowel wall thickening, pathologic Limberg score, and other pathologic ultrasound findings (mesenteric inflammation, lymph nodes, ascites, stenosis, stenosis, fistula, and abscesses) was higher when histopathological inflammation was present (Table 2). The Limberg score was statistically significantly different between patients with histological inflammation and those without. In the absence of histological inflammation, all ultrasound evaluations resulted in a Limberg score of ≤ 2. The diagnostic values of ultrasound-measured bowel wall thickening (> 3 mm and > 4 mm) and Limberg score (≥ 1 and ≥ 2) are shown in Tables 3, 4, and 5. A PPV of > 90% was found for bowel wall thickening and a pathologic Limberg score. A higher threshold for pathologic bowel wall thickness (> 4 mm rather than > 3 mm) leads to better specificity (85.3%), but a worse sensitivity (which drops from 70.1 to 56.1%), compared to > 3 mm threshold. A Limberg score of ≥ 2, indicating the hypervascularization of (neo)terminal ileitis of CD only, has low sensitivity (49.7%) and NPV (24.8%), but the highest specificity (91.2%) and PPV (96.9%) in comparison to thresholds for bowel wall thickening (> 3 mm or > 4 mm) or Limberg ≥ 1. ROC analyses for the detection of histopathological ileal inflammation by bowel wall thickening and by the Limberg score are shown in Figs. 5 and 6, respectively.

**Table 3:** Diagnostic of histopathological ileal inflammation by ultrasound bowel wall thickening

Ileal histology/ultrasound	Bowel wall thickening		
	> 3 mm	≤ 3 mm	*n*
(a)			
Inflammation	131	56	187
No inflammation	9	25	34
*N*	140	81	221

	Bowel wall thickening		
Ileal histology/ultrasound	> 4 mm	≤ 4 mm	*n*
(b)			
Inflammation	105	82	187
No inflammation	5	29	34
*n*	110	111	221

Threshold of 3 mm (a), and 4 mm (b)

**Table 4:** Diagnosis of histopathological ileal inflammation by the Limberg score

Ileal histology/hypervascularization	Limberg ≥ 1	Limberg 0	*n*
(a)			
Inflammation	136	51	187
No inflammation	9	25	34
*n*	145	76	221

Ileal histology/hypervascularization	Limberg ≥ 2	Limberg ≤ 1	*n*
(b)			
Inflammation	93	94	187
No inflammation	3	31	34
*n*	96	125	221

Threshold of ≥ 1 (a) or ≥ 2 (b)

**Table 5:** Accuracy indices of ileal bowel wall thickening and Limberg score for detection of histopathological inflammation (*n* = 221 examinations)

Ileal ultrasound	Sensitivity (%)	Specificity (%)	PPV (%)	NPV (%)
Bowel wall thickness > 3 mm	70.1	73.5	93.6	30.9
Bowel wall thickness > 4 mm	56.1	85.3	95.5	26.1
Limberg ≥ 1	72.7	73.5	93.8	32.9
Limberg ≥ 2	49.7	91.2	96.9	24.8

*PPV* positive predictive value, *NPV* negative predictive value

## Discussion

Bowel wall ultrasound is the first choice in the diagnosis and follow-up of patients with inflammatory bowel disease. Bowel wall thickening and degree of vascularization by the Limberg score correlate (sometimes weakly) with clinical, biochemical or endoscopic disease activity, treatment outcome, and risk of surgery [17–24]. The treatment aim of ‚mucosal healing ‘as well as the significance of histopathological inflammation is debated [7, 23, 25, 26]. Bowel wall thickness (> 3 mm) and color Doppler vascularization are the best predictors of disease activity (apart from the presence of mesenteric inflammatory fat, lymph nodes or complications) [22].

As active CD presents regularly within the terminal ileum (> 66%) [27], ultrasound assessment focuses primarily on this location. In our cohort of 221 bowel wall ultrasound examinations in CD patients taken from an ultrasound register, we found an association of ileal bowel wall thickening and Limberg score with the presence of histological inflammation. Ultrasound findings of bowel wall thickening > 3 mm or hypervascularization (Limberg ≥ 2) indicate histopathological inflammation within the terminal or neoterminal ileum, whereas normal bowel wall thickness (≤ 3 mm) or no hypervascularization cannot exclude histopathological inflammation. A cut-off of > 3 mm for bowel wall thickening leads to higher sensitivity for the presence of histological inflammation, whereas a cut-off of > 4 mm shows a better specificity. Mesenteric inflammation, lymph nodes, ascites, stenosis, and fistula were more frequent in presence of histological inflammation.

In a retrospective analysis, Drews et al. evaluated results of ileocolonic biopsy and ileal power Doppler sonography within 5 days in 32 patients with CD [7]. Using a cut-off of > 4 mm for bowel wall thickening, hypervascularization by the Limberg score indicated active disease in 50% of patients, and histological findings revealed active inflammation in 59% (19/32), whereas 16% had chronic and 25% no inflammation. A sensitivity of 95%, a specificity of 69%, PPV of 90%, and NPV of 82% were calculated for a Limberg score ≥ 1 to detect histological inflammation and 68%, 77%, 63%, and 81% for a Limberg score ≥ 2, respectively. Clinically assessed disease activity showed an association with the Limberg score (*p* = 0.013), but not with histology (*p* = 0.248). Disease duration and body mass index did not influence disease activity. The results of our study, which had a higher number of ultrasound examinations, seems to be comparable with the results in the analysis by Drews et al. In our study, the factor “hypervascularization” (Limberg score ≥ 2) led to lower sensitivity, but higher specificity than seen in Drews et al. Our different cut-off for bowel wall thickening and the fact that any kind of inflammation signs on histology was rated as inflammation, may account for lower sensitivity and NPV in our cohort.

In a 4-year-long study, Sasaki et al. compared results of ultrasound examinations of the small intestine using the Limberg score with histopathological analysis of surgically resected specimens in 10 patients with CD [28]]. Patients with a histopathological grade 3 and 4 Limberg score had significantly higher bowel wall vascularity and inflammatory cell infiltration than those with a low Limberg score. No association between Limberg score and clinical activity staging could be found. Preoperative color Doppler ultrasound was concluded to predict macroscopic and microscopic tissue inflammation in the small intestine of CD patients. Results of studies comparing ultrasound findings with results of ileal biopsies suffer from a selection bias on the part of the endoscopist who decides to take biopsies or not. The endoscopist observing the mucosal surface tends to be more sensitive in detecting inflammation than ultrasound but may also miss microscopic inflammation.

Although our study included an analysis of a high number of ultrasound investigations, the power of the results is limited by certain weaknesses. The retrospective nature of the study has limited the availability of information on exact bowel wall thickness for the normal ultrasound findings. In particular, there is a tendency to a bias towards not taking biopsies when there is no visible inflammation macroscopically during colonoscopy. In consequence, this could have caused an overestimation of false-negative ultrasound examinations. Possible treatment changes during the delay between biopsies of colonoscopies and ultrasound may have an impact in certain cases, although the median time delay is only 8 days. Missing details of the medical history such as HBI or the exact treatment regimen in some patients may have led to a bias. Detection of hypervascularization may depend on technical settings of the ultrasound machines, depth of the ileum and degree of overweight.

The detection of small vessels might have been missed with higher pulse repetition frequencies when using the color Doppler. Moreover, the classification of the Limberg score could be arbitrary to a degree. The proportion of ultrasound examinations in patients during outpatient visits is high (73%), leading to a certain selection bias and representing the changes in disease burden in patients with inflammatory bowel disease due to the availability of novel pharmaceutical products. Because current treatment strategies can reduce disease activity significantly, new imaging modalities are underway for the non-invasive assessment in this group of patients [28, 29]. Examinations in patients with higher disease activity might have shown more significant results, but would not be representative for most of the patients today. Nevertheless, trends could be identified. The presence of histological inflammation on a microscopic level is difficult to predict by ultrasound in the present cohort, treated with modern treatment options.

In conclusion, pathologic ultrasound findings such as bowel wall thickening, elevated Limberg score, and bowel surrounding peculiarities are frequently found in our CD cohort. Bowel wall thickening (> 3 mm) and a pathologic Limberg score are good predictors of histopathological inflammation within the terminal or neoterminal ileum. Normal ultrasound findings without bowel wall thickening and without hypervascularization do not rule out inflammation in histopathological analysis.
